# Clinical Trials Supporting the Role of the IL-17/IL-23 Axis in Axial Spondyloarthritis

**DOI:** 10.3389/fimmu.2021.622770

**Published:** 2021-06-02

**Authors:** Angela Ceribelli, Francesca Motta, Matteo Vecellio, Natasa Isailovic, Francesco Ciccia, Carlo Selmi

**Affiliations:** ^1^ Division of Rheumatology and Clinical Immunology, Humanitas Clinical and Research Center - IRCCS, Rozzano (Mi), Italy; ^2^ Department of Biomedical Sciences, Humanitas University, Rozzano (Mi), Italy; ^3^ Nuffield Department of Orthopaedics, Rheumatology and Musculoskeletal Sciences, University of Oxford, Oxford, United Kingdom; ^4^ Università degli Studi della Campania Luigi Vanvitelli, Naples, Italy

**Keywords:** Th17, enthesitis, spondylitis, biologics, HLA B27 allele

## Abstract

The term spondyloarthritis (SpA) encompasses a heterogeneous group of inflammatory musculoskeletal diseases with several common genetic background and clinical features, including the possible involvement of the axial skeleton with peripheral mono- or oligo- arthritis and frequently coexisting skin, eye and intestinal manifestations. When the sacroiliac joints or other parts of the spine or thoracic wall are predominantly affected at magnetic resonance or X-ray imaging with inflammatory back pain, the disease is classified as axial SpA and the therapeutic choices are significantly different compared to cases of peripheral arthritis. Moving from the narrow effectiveness and safety profiles of non-steroidal anti-inflammatory drugs, there has been a significant research effort aimed at identifying new treatments based on our better understanding of the pathogenesis of SpA. Indeed, in parallel with the solid data demonstrating that IL-17 and IL-23 are key cytokines in the development of enthesitis and spondylitis, monoclonal antibodies interfering with this pathway have been developed for the treatment of axial SpA. Furthermore, the IL-17/IL-23 axis is key to extra-articular manifestations such as inflammatory bowel disease, uveitis, and psoriasis which are frequent comorbidities of SpA. Currently available drugs act through these mechanisms recognizing IL-23 and targeting IL-17, such as secukinumab and ixekizumab. These therapeutic approaches are now envisioned in the international treatment recommendations for psoriatic arthritis with an axial phenotype as well as for ankylosing spondylitis (AS). We will provide herein a concise comprehensive overview of the clinical evidence supporting the use of these and other drugs acting on IL-23 and IL-17 in axial SpA.

## Introduction

The role of interleukin (IL)-17 and IL-23 in the pathogenesis of chronic inflammatory diseases such as spondyloarthritis (SpA) has been widely investigated over the past two decades, and this knowledge has led to the development of targeted therapies (1). IL-17 was first described in 1996 due to its effect on the production of IL-6 and IL-8 by rheumatoid arthritis (RA) synoviocytes (2) and the new cytokine was first named IL-17A but several other members of the family were subsequently identified, in particular IL-17F, which is approximately 50% homologous to IL-17A and both converge on TNFα, among other mediators (3, 4).

**Figure 1:**
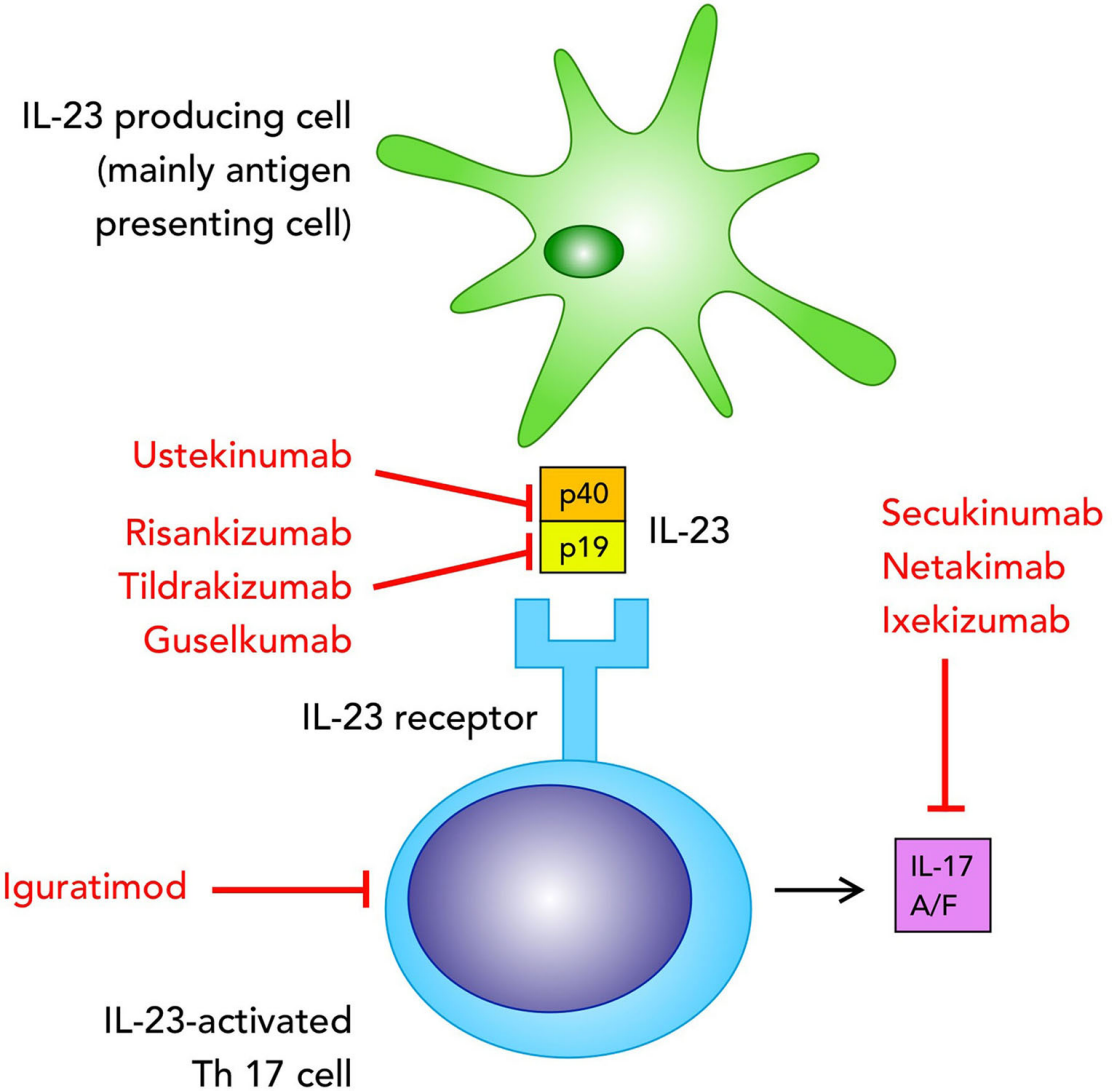
Biologics targeting the IL-23/IL-17 pathway in axial spondyloarthritis, currently used in clinical practice or tested in clinical trials mentioned in the text.

IL-23 induces IL-17A, IL-17F, IL-21 and IL-23 production (5, 6) thus playing an important role in the Th17 cell-mediated responses. IL-23 is part of the family of IL-12 cytokines and IL-12 and IL-23 share a common p40 subunit, coupled with a p35 chain for IL-12 and a p19 chain for IL-23 (7). IL-17 is a product of T cells, particularly Th17, even though several other cell types are able to produce IL-17, such as CD8+ T cells, γδ T cells, type 3 innate lymphoid cells (ILC3s) and natural killer T cells (8, 9). Other cell types such as neutrophils and mast cells do not express IL-17 mRNA but they can store exogenous IL-17 (10, 11). On the other hand, both IL-12 and IL-23 are produced by antigen-presenting cells, in particular dendritic cells, monocytes and macrophages, and based on the predominant presence of these cells in the inflamed tissues we can predict how the contribution of IL-23 is significant in a specific disease (5, 7) (**Figure 1**).

Both IL-23 and IL-17 have shown a significant therapeutic effect first in animal models and subsequently in patients affected by rheumatic conditions such as axial SpA (axSpA), i.e. a form of SpA that predominantly affects the axial skeleton, ranging from sacroiliitis to paradigmatic AS (1, 12). In particular, IL-17 inhibitors are currently approved as biological disease-modifying anti-rheumatic drugs (DMARDs) for axSpA, together with TNFα inhibitors, and currently include secukinumab [a human IgG1κ monoclonal antibody that binds to IL-17A (13)], ixekizumab [a humanized monoclonal antibody anti-17A (14)], while bimekizumab [a humanized monoclonal antibody anti-17A and IL-17F (15)] and netakimab [a humanized monoclonal antibody targeting interleukin-17A (16)] are being evaluated. On the other hand, IL-12/IL-23 inhibitors are effective against psoriasis (PsO) and psoriatic arthritis (PsA), predominantly on peripheral synovitis and enthesitis in axSpA (1), while the efficacy on axial manifestations remains inconclusive.

All biologic therapies currently approved for the treatment of axSpA are included in the recent recommendations approved in 2019 by the American College of Rheumatology, Spondylitis Association of America and Spondyloarthritis Research and Treatment Network (17), which strongly recommend to treat adult patients with active AS despite TNFα inhibitor (primary non-responder) with secukinumab or ixekizumab, while IL17 inhibitors are not recommended in the presence of inflammatory bowel disease (IBD) or recurrent uveitis.

In this concise review we will discuss the available clinical trials data and the recent advances in the discovery of new therapies for axSpA, with a specific focus on therapies targeting the IL-17/IL-23 axis.

## Clinical Trials in axSpA for Available Drugs Targeting the IL-17A/IL-23 Axis

In the last 5 years, new therapies for the treatment of axSpA have been approved thanks to the significant positive results in efficacy and safety obtained by these therapies targeting the IL-17/IL-23 axis.

### Anti IL-17A Drugs

Two anti-IL-17A monoclonal antibodies, secukinumab and ixekizumab, are currently approved for the treatment of axSpA.

Secukinumab was initially approved for PsO treatment, later for PsA and axSpA. It was demonstrated that, compared with placebo, a significantly higher percentage of patients with active PsA achieved a 20% improvement in the American College of Rheumatology response criteria (ACR20) at week 24 when treated with secukinumab (18, 19). It provided a valid treatment for PsA entheseal disease (20), and was effective for signs and symptoms of axial disease in PsA and SpA patients. MAXIMISE evaluated the efficacy and safety of secukinumab (at the dosage of 300 and 150mg) in managing the axial manifestations of PsA, and results showed a rapid and significant improvement in ASAS20 responses at week 12 for axial manifestations and inadequate responses to NSAIDs (21) (ClinicalTrials.gov Identifier: NCT02721966). More recently, secukinumab proved efficacious for the treatment of non-radiographic axSpA (US FDA, June 2020) based on the results obtained from the PREVENT clinical trial (ClinicalTrials.gov Identifier: NCT02696031). By further searching the site clinicaltrials.gov for interventional studies in phase III aimed at blocking IL-17 in axSpA, we retrieved 7 studies focused on the use of secukinumab, for the analysis on the benefit of this drug on symptoms such as pain and on its safety in a 3-year follow-up period. One of these trials is focused on the speed of secukinumab-induced relief from pain in patients with axSpA (SKIPPAIN trial, ClinicalTrials.gov Identifier: NCT03136861), and its action on specific clinical manifestations such as axial involvement. Besides evaluating the efficacy of this drug, also its safety and tolerability are under evaluation for a follow-up period up to 3 years after marketing secukinumab.

Ixekizumab demonstrated efficacy for the treatment of moderate to severe plaque PsO since 2016. The indication was approved in 2017 for PsA, based on the reported ACR20 in a higher proportion of patients when compared with placebo (22) and showing efficacy also on entheseal disease (23). In 2019 it was approved for active radiographic axSpA thanks to its efficacy and safety outcome results (24, 25). More recently, ixekizumab was evaluated in the COAST-X trial in non-radiographic axSpA patients and it showed superiority to placebo at weeks 16 and 52, with similar rate of adverse events compared with previous ixekizumab studies. Results show that ixekizumab may be a therapeutic option for patients with non-radiographic axSpA who did not respond significantly or were intolerant to NSAIDs (24, 26), and this led to the U.S. FDA approval for the treatment of non-radiographic axSpA in June 2020.

Netakimab (BCD-085) is a novel molecule currently in phase III clinical trials in axSpA patients. In a Phase I clinical trial (ClinicalTrials.gov Identifier: NCT02380287), netakimab was evaluated for pharmacokinetics and safety in healthy volunteers, and results reported in October 2015. Thanks to the data from the Phase I trial, a randomized, placebo-controlled, double-blind Phase II trial (ClinicalTrials.gov Identifier: NCT02763111) was started to evaluate safety, effectiveness, and pharmacokinetic profile in 89 men and women with SpA. This drug was tested at doses of 40 mg, 80 mg, or 120 mg for 12 weeks and the trial results showed significant improvements in AS assessment score (ASAS), plus adverse events, withdrawal symptoms, and drug concentration. The results of this phase II trial (16) showed that netakimab was effective and generally safe in SpA patients, thus it was followed by the multicenter, randomized, placebo-controlled Phase III clinical trial (ClinicalTrials.gov Identifier: NCT03447704) which evaluated the safety and effectiveness of netakimab 120 mg against a placebo, for up to one year, in 228 patients with active SpA. Netakimab has been registered in spring 2020 for treatment of SpA and PsA in Russia. According to the Phase III clinical study BCD-085-5/ASTERA, 40% of patients with SpA achieve an ASAS40 response after 16 weeks of therapy (27).

A recent publication by Mease et al. showed the results of two phase III trials (AMVISION-1 and AMVISION-2) on the efficacy and safety of brodalumab, an IL-17 receptor subunit A inhibitor, compared with placebo, in patients with PsA (28). Results show that brodalumab confers a rapid and significant improvement in signs and symptoms of PsA versus placebo and it has a tolerance and safety profile similar to other IL-17 inhibitors (28).

Iguratimod (IGU), a novel small molecule with the effect similar to a non-steroidal anti-inflammatory drug and disease-modifying anti-rheumatic drug, acts through various mechanisms such as inhibition of prostaglandin E2, TNFα, IL-17 production, macrophage migration inhibitory factor (MIF)- induced proinflammatory effects, osteoclastogenesis, and it promotes osteoblastic differentiation. IGU may be an effective treatment for axSpA as its mechanisms of action are related to those involved in the pathogenesis of axSpA, as shown in several small-scale clinical trials (29). IGU may be a suitable treatment for axSpA as clinical trials demonstrate a significant improvement for the ASAS20 response and reduction in inflammatory biomarkers in patients receiving IGU (29, 30).

### Anti IL-23 Drugs

Ustekinumab, a human IL-12 and IL-23 antagonist that binds to their p40 subunit so that they subsequently cannot bind to their receptors to trigger pro-inflammatory cytokine release, is currently approved for use in moderate-severe PsO, PsA and Crohn’s disease, while its efficacy in the treatment of axSpA was not demonstrated in 3 placebo-controlled trials, although its safety profile was consistent with studies in other indications (31). More recently, ustekinumab has been evaluated for the use in adult patients affected by moderate-severe ulcerative colitis, while its use failed in other autoimmune diseases such as multiple sclerosis (32), and encouraging data were provided in systemic lupus erythematosus.

Guselkumab, a monoclonal antibody targeting the IL-23 subunit alpha (p19 subunit) currently approved for the use in plaque PsO, is under evaluation for the use also in PsA with significant improvement in particular for manifestations such as enthesitis and dactylitis (33). The results of two phase III, randomized, double-blind, placebo-controlled studies (DISCOVER-1 and -2) on the efficacy of guselkumab in active PsA patients showed a significant improvement of the disease with ACR achievement over 24 weeks (34, 35).

Risankizumab is a monoclonal antibody that binds the p19 subunit of IL-23 and demonstrated efficacy in PsO and active peripheral PsA in preapproval trials (36), while a phase II study in axSpA did not show statistical difference from placebo and for this reason the further development of risankizumab in axSpA was not continued (37).

In February 2020, tildrakizumab, a monoclonal antibody targeting the IL-23 p19 subunit, was approved in Italy for treatment of severe plaque PsO thanks to the significantly positive results obtained in the reSURFACE 1 and 2 trials (22, 23). Efficacy and safety of tildrakizumab is under evaluation also for the treatment of active AS or non-radiographic axSpA (ClinicalTrials.gov Identifier: NCT 02980705). A phase II trial based on the use of tildrakizumab, is currently ongoing and results are still being analyzed, but the failure of two previous studies in axSpA with drugs with similar mechanism of action raises doubts these ongoing studies will be successful for treatment in axSpA (38).

Clinical trials focused on the inhibition of IL-23/IL-17 have been fundamental in PsO, while they showed mixed results in PsA and were even less conclusive in axSpA (39) (**Table 1** summarizes IL-17/IL-23 blocking molecules studied in clinical trials for the treatment of axSpA. **Figure 1** for their mechanisms of action). These results may mean that we still need to improve our knowledge of SpA, in particular for its pathogenesis, thus further head-to-head studies and more subtle evaluations of local tissue-specific mechanisms are required.

**Table 1:** Molecules blocking the IL-17/IL-23 axis, studied for the treatment of axSpA.

Drug	Target	Disease (clinical trial)	Cohort analyzed	Results
Secukinumab	Monoclonal antibody binding IL-17A	PsA	Enrolled 996 active PsA	Efficacy and safety on sign and symptoms of active PsA and entheseal disease
		(FUTURE, phase III)	Mean age: 48y	
			F: 49%	
			No prior anti-TNF therapy: 70%	
			Enthesitis: 60% at baseline	
		axPsA	Enrolled 498 active axPsA	Rapid and significant improvement in ASAS20 responses at week 12 for axial manifestations
		(MAXIMISE, phase III)	Mean age: 46y	
			F ≈48%	
			No prior bDMARD: 100%	
			Enthesitis: SPARCC score ≈4.6 at baseline	
		nr axSpA	Enrolled 555 nr axSpA with sacroiliac inflammation at MRI	Improvement compared to placebo-treated patients at week 16 for general health status, quality of life and safety
		(PREVENT, phase III)	Mean age 39y	
			F ≈54%	
			No prior anti-TNF therapy: 90%	
			History of IBD ≈2%	
		axSpA (SKIPPAIN, phase III)	Enrolled 383 active axSpA	Efficacy, safety, pain relief, improvement of axial disease
			Mean age 42y	
			F 38%	
Ixekizumab	High-affinity IL-17A monoclonal antibody	PsA	Enrolled 363 active PsA	Improvement in the signs and symptoms of patients with active PsA and entheseal disease
		(SPIRIT-P1 and -P2, phase III)	Mean age ≈51y	
			F ≈53%	
			Previously treated with anti-TNFα with inadequate response or intolerant to anti-TNFα: 100%	
			Enthesitis: ≈60% at baseline	
		nr axSpA	Enrolled 303 active nr axSpA	Efficacy
		(COAST-X, phase III)	Mean age 40y	
			F ≈52%	
			No prior anti-TNFα therapy: 100%	
			Enthesitis: ≈48% at baseline	
			IBD: ≈1%	
Netakimab (BCD-085)	Monoclonal antibody blocking IL-17	Registered for treatment of AS and PsA in Russia, from spring 2020	Phase II: enrolled 89 active AS	Effective and generally safe
			Mean age ≈38y	
			F ≈17%	
			No prior anti-TNFα therapy: ≈85%	
			Phase III: enrolled 228 active AS, no results posted	
Brodalumab	Monoclonal antibody against IL-17 receptor A	PsA	Enrolled 962 active PsA	Improvement in signs and symptoms of PsA versus placebo, safety profile similar to other IL17 inhibitors
		(AMVISION-1 and -2, phase III)	Mean age ≈48y	
			F ≈50%	
			No prior bDMARDs: 70%	
			Enthesitis: ≈67% at baseline	
Iguratimod	Inhibitor of IL-17 production and additional mechanisms	AxSpA	4 RCTs and 2 case series	Significant improvement for the ASAS20 response and reduction in inflammatory biomarkers
		(small-scale clinical trials)	Active or refractory axSpA	
Bimekizumab	Monoclonal antibody against IL-17A and IL-17F	AS	Enrolled 303 active AS	Significant improvement for the ASAS40 response at week 12
		(Phase IIb clinical trial)	Mean age ≈40y	
			F ≈15%	
			No prior bDMARDs: ≈90%	
Guselkumab	Monoclonal antibody binding the p19 subunit of IL-23	PsA	DISCOVER-1: enrolled 381 active PsA	Rapid and significant improvement in PsA patients biologic-naive or previously treated with TNFα inhibitor treatment
		(DISCOVER-1 and -2, phase III)	Mean age ≈48y	
			F 48%	
			No prior use of bDMARDs: 69%	
			Enthesitis: ≈58% at baseline	
			DISCOVER-2: enrolled 741 active PsA	
			Mean age ≈46y	
			F ≈47%	
			No prior use of bDMARDs: 100%	
			Enthesitis: ≈68% at baseline	
Risankizumab	Monoclonal antibody that inhibits IL-23 by binding to its p19 subunit	AS	Enrolled 159 active AS	No statistical difference from placebo
		(Phase II clinical trial)	Mean age ≈38y	
			F ≈28%	
			No prior use of bDMARDs: 100%	
Ustekinumab	Monoclonal antibody blocking the p-40 subunit of IL-12/IL-23	AxSpA	Enrolled 1018 active AS and nr axSpA	No demonstration of efficacy
		(Phase III clinical trials)	Mean age ≈38y	
			F ≈73%	
			No prior use of bDMARDs: 100% in studies 1 and 2, 88% in study 3.	
Tildrakizumab	Monoclonal antibody that inhibits IL-23 by binding to its p19 subunit	AS, nr axSpA	Enrolled 180 active AS or nr axSpA	Failure of two previous studies with drugs with similar mechanism of action; efficacy and safety under evaluation in SpA
		(Phase IIa clinical trial)	Mean age 39y	
			F ≈23%	

AxSpA, axial spondyloarthritis; axPsA, psoriatic arthritis with axial involvement; nr axSpA, non-radiographic axial SpA; AS, ankylosing spondylitis. ASAS, Assessment of Spondyloarthritis international Society. SPARCC, Spondyloarthritis Research Consortium of Canada enthesitis index. DMARD, disease-modifying anti-rheumatic drug. y, years. F, female.

In light gray, effective drugs; in black, non effective drugs; in dark gray, ongoing study.

## Clinical Trials in axSpA for Available Drugs Targeting IL-17F

IL-17A shares a structural homology with IL-17F (55%) and has been reported to perform a similar biological function. Depending on the nature of the responder cell, the ligation of IL-17R triggers signaling pathways causing the activation of the transcription factors NFκB, IκBζ, AP1 and C-EBP, which induce transcription of several tissue-specific genes (40). However, recent studies have shown that IL-17F forms a homodimeric complex with receptor IL-17RC driving IL-17RA-independent and therefore (IL-17A-independent) signaling (41). The real distribution of the two IL-17RA and IL-17RC receptors on the surface of the cellular actors involved in the pathogenesis of AS is not clear, but it appears likely that some cell types may overexpress IL-17RC compared to IL-17RA, which could make IL-17F signaling through the symmetrical IL-17RC complex more relevant in some patients. Evidences that IL-17F is also increased in psoriatic skin and synovial cells of patients with peripheral SpA (42), support the rationale for targeting IL-17F as a therapeutic strategy as well.

In a recent phase IIb, randomized, double-blind, placebo-controlled, dose-ranging study, the efficacy of dual neutralisation of IL-17A and IL-17F with bimekizumab in patients with active AS has been studied. At week 12, significantly more bimekizumab-treated patients achieved ASAS40 vs placebo with a significant dose-response. At week 48, 58.6% and 62.3% of patients receiving bimekizumab 160 and 320 mg throughout the study achieved ASAS40, respectively with similar ASAS40 response rates being observed in re-randomized patients. Although these data are absolutely promising, they are not very different from the single inhibition of IL-17A as can be assumed from a theoretical point of view (34). Greater precision in patient selection, trivially assessing the peripheral distribution of IL-17RA and IL-17RC receptors, could increase the percentage of responders. However, further studies are required to determine the impact of double inhibition, IL-17A and IL-17F on the progression of radiographic progression in AS patients.

## Other Mechanisms of Action in axSpA Therapies

A detailed discussion of the clinical efficacy of anti-TNFα goes beyond the aims of the present review article. The current recommendations for AxSpa and PsA include this class of drugs on the same line as other mechanisms of action and based on this TNF inhibitors will be mentioned as comparators, as shown in **Table 2** (43, 44).

**Table 2:** Comparison of different biologic class efficacy in rheumatic diseases and disease subtypes.

	Anti-TNFα	Anti-IL-17	Anti-IL-23	JAK inhibitor	PDE4 inhibitors
**RA**	+	–	–	+	–
**AxSpA**	+	+	–	Ongoing studies	–
Disease activity					
Radiographic progression	+	+	–	Ongoing studies	–
**PsA**	+	+	+	+	+
Enthesitis	+	+	+	+	+
Peripheral arthritis	+	+	+	+	+
Dactylitis	+	+	+	+	+
Axial manifestations	+	+	–	Ongoing studies	–
**Psoriasis**	+	+	+	–	+
Nail	+	+	+	–	+
**IBD**	+	–	+	+	–
**Acute anterior uveitis**	+	–	–	–	–

AxSpA, axial spondyloarthritis; IBD, inflammatory bowel diseases; RA, rheumatoid arthritis; PsA, psoriatic arthritis; PsO, psoriasis.

Besides the anti-TNFα therapies approved in the last 20 years for the treatment of SpA patients (45–47), with proved efficacy also for the treatment of extraarticular manifestations such as uveitis (RAPID-axSpA ClinicalTrials.gov Identifier: NCT01087762) (48), biosimilars of TNFα inhibitors have become increasingly used, and observational studies of biologics-naïve patients with SpA have shown similar response and safety in patients treated with originators versus biosimilars, indicating comparable effects in clinical practice (49).

Oral small molecules targeting specific pro-inflammatory intracellular pathways are currently used in conditions such as PsA, as in the case of the phosphodiesterase 4 inhibitor apremilast, and the Janus kinase (JAK) inhibitors tofacitinib and baricitinib, plus filgotinib and upadacitinib still in development for PsA. The rationale for the use of JAKinibs in AS derives from studies in experimental models that have demonstrated a key role of the JAK/STAT pathway in the pathogenesis of the disease (50, 51). Moreover, *ex vivo* data also demonstrated the ability of several JAK inhibitors to inhibit Th17 responses in patients with AS. The potential efficacy of JAKinibs in the treatment of axial SpA is supported by recent phase II and III clinical trials. Tofacitinib, a JAK1 and 3 inhibitor, was tested in a phase II study in 208 AS patients (52). The ASAS20 response at week 12 occurred in 63%, 67%, and 40% in the tofacitinib 5 and 10 mg arms and placebo, respectively, and reduction of inflammation measured by MRI was demonstrated. The phase III study in AS is currently ongoing (ClinicalTrials.gov Identifier: NCT03502616). Upadacitinib, a selective JAK-1 inhibitor, has been also studied in AS patients with active disease and an inadequate response or contraindication to non-steroidal anti-inflammatory drugs in a double-blind, randomised controlled phase 3 trial, the SELECT-Axis study 1. In this study, significantly more patients in the upadacitinib group reached a higher ASAS40 response compared to placebo group at week 14 (52% *vs* 26%) (53). The TORTUGA study evaluated the efficacy of another selective JAK-1 inhibitor, filgotinib, in patients with AS (54). In this study, at week 12, 76% of patients receiving filgotinib achieved an ASAS20 response compared with 40% of patients assigned to placebo. ASAS40 was achieved by 38% patients assigned to filgotinib and by 19% patients assigned to placebo. Based on these studies, JAK blockade could represent a valid future therapeutic strategy in patients with AS.

## Discussion

From a pathological point of view, IL-23 is a crucial cytokine in the onset of disease manifestations such as enthesitis that may characterize peripheral manifestations in axSpA, as demonstrated by the fact that IL-23 is sufficient to induce the development of enthesitis and entheseal new bone formation in the initial complete absence of synovitis (55). IL-23 stimulates the survival and expansion of Th-17 cells through the receptor IL-23R expressed by uncommitted CD4 and CD8 negative T cells, and this induces the related downstream signaling pathway crucial for the onset of Th-17-mediated diseases like PsA and axSpA (56).

Consistent with these mechanistic models, IL-17 inhibitors showed efficacy in axSpA treatment (13, 14) and case-control genome-wide-association studies demonstrated that an IL-23R polymorphism is associated with SpA (57). Moreover, the overexpression of IL-23 in mice can trigger a form of enthesitis which is similar to enthesitis observed in SpA patients (55). Therefore, it was unexpected to observe the results of two placebo-controlled trials in SpA showing that ustekinumab (31) and risankizumab (37) had no significant improvement on disease activity.

Inconclusive data from trials on IL-23 inhibitors may have several explanations, such as still unknown mechanisms of disease, different molecular effects compared to IL-17 blockers or confounding factors in the design of clinical trials, as heterogeneous enrollment with differences in the composition of the clinical cohorts where the monoclonal antibodies are being investigated (shown in **Table 1**). A possible additional mechanism of action of IL-17 and IL-23 involves bone metabolism, which is a pivotal pathogenic pathway in axSpA, related to inflammation. As for IL-17A, it has a potential effect on osteoblast differentiation that may depend on the cell type exposed, the differentiation stage of that cell and the timing and duration of cytokine exposure. On the contrary, IL-23 does not seem to have an effect on osteoblast activation (58).

Another difference in the molecular effects of the two cytokines is related to the timing, as IL-23 is thought to play a role in the early stages, making its blocking ineffective in established and symptomatic disease. In murine models the presence of IL-23 induced the conversion of non-pathogenic Th17 into pathogenic Th17 cells, an effect still to be well analyzed in humans (58).

The hypothesis of a non-linear relationship and an uncoupling of IL-23 and IL-17 can also be inferred from Crohn’s diseases management, where IL-23 inhibition has some efficacy, while IL-17 blockade can worsen the outcomes.

A deeper knowledge of the IL-17/IL-23 axis should be pursued, as it appears to be one of the main pathways involved in the development of axSpA. In fact, several therapies currently used in conditions such as RA have not demonstrated efficacy in axSpA, as in the case of IL-1 or IL-6 inhibitors, T-cell modulators and B-cell ablators. Furthermore, apremilast, introduced in the recommendation for the management of peripheral PsA (43) was tested for active AS in a small proof-of-concept study (59), showing improvement of BASDAI in the treatment versus placebo arm, but the phase III trial failed to discriminate between treated and placebo patients (POSTURE study, ClinicalTrials.gov Identifier: NCT01583374). These results show that it is not enough to reduce inflammation in synovial tissues by using unspecific drugs, and that immunomodulatory agents do not all reach the target to reduce inflammation, in particular in a condition such as axSpA which involves many immunologic pathways in different anatomical areas and where inflammation is related to bone formation in specific sites. Additional chronic autoimmune diseases related to axSpA, such as PsA and IBD- related arthritis are currently evaluated to identify new therapeutic pathways and targets that may lead to disease remission.

Another significant aspect that we may consider for trials results analysis is that AS and axial PsA may be two different diseases with overlapping features rather than entities on the spectrum of the same disease (60, 61). In fact, AS patients with or without PsO seem to be consistently different from axSpA patients when it comes to their demographic features (15 years younger at their first manifestation of arthritis and first presented to the clinic 7 years earlier), genetic predisposition (much higher male predominance and four times more likely to be HLA-B27 positive), clinical characteristics (worse axial disease in AS versus axial PsA, whereas axial PsA has worse peripheral arthritis than AS patients) and radiographic alterations (worse grade of sacroiliitis on radiographs).

These aspects are crucial in the planning of clinical trials, when defining the population of patients to be included, as it may influence the results of specific treatments.

When choosing the biologic therapies for SpA patients, IL-17 inhibitors have always raised concern over the risk of IBD onset. In fact, the clinical development of secukinumab for Crohn’s disease was stopped after a small sample size proof-of-concept study because of a higher rate of adverse events including relapse of pre-existing or new onset of IBD (62), and similarly in the phase-2 trial with brodalumab (63). The concern over elevated risk of IBD onset or worsening was minimized in a recent pooled safety analysis of 21 randomised-controlled trials plus post-marketing safety data (2014–2017) with secukinumab, across all rheumatological indications. This analysis reported an exposure adjusted incidence rate of IBD of <0.1- 0.4/100 person-years consistent with the background expected range of incidence rates for these patients (64–66). In conclusion, clinical trials have so far demonstrated the efficacy of anti-IL-17 therapies for axSpA treatment but data on IL-23 blocking remain to be elucidated after the earlier negative findings. This may be due to several reasons, such as the unique immunopathological microenvironment of axSpA, with IL-17 secretion in the absence of IL-23, at least in established disease. Another possible reason may be that many cell types different from conventional T cells can produce IL-17 in axSpA patients, partially independent of IL-23, and this should be extensively investigated. Currently, IL-17 and TNFα inhibitors are the only effective targeted therapies for axSpA and additional treatments need further testing in clinical trials to assess their efficacy and safety in axSpA.

## Author Contributions

AC and FM contributed to data collection and review organization. NI and MV contributed to finalizing the review writing. FC and CS supervised the review writing and organization. All authors contributed to the article and approved the submitted version.

## Conflict of Interest

The authors declare that the research was conducted in the absence of any commercial or financial relationships that could be construed as a potential conflict of interest.
